# A Service Reconfiguration Bundle for Expanding Access to Peritoneal Dialysis Including for Older Frailer Patients

**DOI:** 10.3390/healthcare11111654

**Published:** 2023-06-05

**Authors:** Michael Corr, Carolyn Hunter, Daniel Conroy, Damian McGrogan, Damian Fogarty, Stephen O’Neill

**Affiliations:** 1 Centre of Public Health, Queen’s University Belfast, Belfast BT7 1NN, UK; 2 Regional Nephrology & Transplant Unit, Belfast Health and Social Care Trust, Belfast BT9 7ER, UK; 3 Nephrology Department, Northern Health and Social Care Trust, Antrim BT41 2RL, UK; 4 Interventional Radiology Department, Belfast Health and Social Care Trust, Belfast BT12 6BA, UK

**Keywords:** peritoneal dialysis, service reconfiguration, renal replacement therapy, frailty

## Abstract

Introduction: Rates of peritoneal dialysis (PD) have been traditionally low in Northern Ireland. With rising numbers of patients reaching end-stage kidney disease, PD is a more cost-effective treatment than haemodialysis and aligns with international goals to increase home-based dialysis options. The aim of our study was to highlight how a service reconfiguration bundle expanded access to PD in Northern Ireland. Methods: The service reconfiguration bundle consisted of the appointment of a surgical lead, a dedicated interventional radiologist for fluoroscopically guided PD catheter insertion, and a nephrology-led ultrasound-guided PD catheter insertion service in an area of particular need. All patients in Northern Ireland who had a PD catheter inserted in the year following service reconfigurations were included and prospectively followed up for one-year. Patient demographics, PD catheter insertion technique, setting of procedure, and outcome data were summarised. Results: The number of patients receiving PD catheter insertion doubled to 66 in the year following service reconfigurations. The range of approaches to PD catheter insertion (laparoscopic *n* = 41, percutaneous *n* = 24 and open *n* = 1) allowed a wide range of patients to benefit from PD. Six patients had emergency PD catheter insertion, with four receiving urgent or early start PD. Nearly half (48%, 29/60) of the PD catheters inserted electively were in smaller elective hubs rather than the regional unit. A total of 97% of patients successfully started PD. Patients who experienced percutaneous PD catheter insertion were older [median age 76 (range 37–88) vs. 56 (range 18–84), *p* < 0.0001] and had less previous abdominal surgery than patients who experienced laparoscopic PD catheter insertion (25%, 6/24 vs. 54%, 22/41, *p* = 0.05). Discussion: Through a service reconfiguration bundle, we were able to double our annual incident PD population. This study highlights how flexible models of service delivery introduced as a bundle can quickly deliver expanded access to PD and home therapy.

## 1. Introduction

Chronic kidney disease (CKD) is increasingly prevalent as populations age, and underlying diseases, such as diabetes and hypertension, that lead to CKD become more common [1]. By 2040, CKD is expected to be the fifth leading cause of death worldwide, rising from 12th in 2017 [2]. As the prevalence of CKD increases, more patients are reaching end-stage kidney disease (ESKD) and require renal replacement therapy (RRT). Whilst many patients proceed to receive a kidney transplant (the gold-standard RRT), the number of patients receiving dialysis treatment is rising in most countries [3]. The burden of CKD is particularly high in those within the lowest quintiles of socio-demographic indices and in low- and middle-income countries.

The two most common outpatient dialysis modalities are intermittent haemodialysis (HD) and peritoneal dialysis (PD). Studies have failed to clearly demonstrate any survival benefit between the two therapies but this is thought to be due lack of randomised studies, selection/intention to treat biases, time-from-initiation bias, and comorbidity difference in studied populations [4]. However, PD has some advantages over HD. It is often regarded as a gentler dialysis therapy that preserves native urine output; hence, it is recommended by guidelines as a bridging RRT for patients awaiting transplantation, and most importantly, it has major advantages for resource-poor countries or those with large distances between population and renal units [5]. It can be a useful modality in frailer patients who are less able to tolerate the haemodynamic instability associated with HD [6]. PD also preserves vascular access (required for HD), which can be beneficial in younger patients who face a lifetime of RRT [7]. Patients on PD often report having a higher quality of life than those on HD, and the flexibility of PD can help patients continue their education or work with minimal disruption [8]. Due to the reduced hospital interactions of PD patients versus HD, PD is associated with reduced healthcare costs and can assist renal units in managing increasing demand for HD outpatient slots [9,10]. Hence, whilst survivability benefits compared to HD remain debatable, PD has significant benefits on other outcomes and metrics that are important to both patients and clinicians [11]

PD use in Northern Ireland (NI) has traditionally been lower than the rest of the United Kingdom (UK) (49 per million population vs. 72 per million population in England in 2019) [12]. Whilst the discrepancy is partially attributable to a successful transplant programme (prevalent transplant population NI 884 per million population vs. 735 per million population in UK) [12], lack of access to PD catheter insertion has limited its prompt availability as a dialysis modality.

Optimal service delivery for PD catheter insertion and wider PD programmes are underrepresented within the literature [13]. Clinician attitudes to PD can affect the uptake of the RRT modality and be a barrier for the expansion of PD services [14]. Reduced ability for frailer patients to self-care, contraindications to catheter insertion (especially with open PD catheter insertion technique), and delays in PD access creation can also limit the uptake of PD [15]. Successful PD programmes require the careful selection of patients, various access options despite comorbidities, and the ability to manage the increasing frailty in the ESKD population [13]. They must also have the capacity to meet demand with minimal delay to the individual patient’s journey [16]. Finally, a successful programme should involve collecting data on its outcomes for quality improvement and maintenance purposes. This can facilitate promoting PD by sharing success and enable adequate resource provision to be secured by highlighting funding requirements [13].

In 2021, understanding the need to expand access to PD for our patients, we bundled a range of service reconfigurations to help diversify and expand access to PD across NI. The service reconfiguration bundle consisted of the appointment of a surgical lead, a dedicated interventional radiologist for fluoroscopically guided PD catheter insertion, and a nephrology-led ultrasound-guided PD catheter insertion service in an area of particular need. The aim of this study was to describe and assess our regional experience of expanding access to PD through a service reconfiguration bundle for PD catheter insertion.

## 2. Methods

### 2.1. Service Offered Prior to Reconfiguration

This study was carried out in NI, a region of the UK with a population of 1.9 million across 14,130 km^2^ (density 135/km^2^). As a region, it has an annual incidence of ~205 patients commencing RRT across five hospital trust sites, each with a HD unit but one central transplant site [12]. Prior to service reconfiguration, PD access procedures were performed for the whole NI population via a surgical service at the Regional Nephrology and Transplant Centre. Previously all PD catheter insertions in NI were performed using an open surgical technique, usually under general anaesthesia, in elective operating theatres in the Regional Centre. Antrim Area Hospital, which is the dialysis unit for the Northern Trust, was one of the main referring hospitals for PD access procedures but did not offer an insertion service. The Northern Trust has a particularly large and rural geographical distribution that makes home therapy, such as PD, highly desirable for the population served.

### 2.2. Service Reconfiguration Bundle

A surgical lead for peritoneal dialysis was appointed, and laparoscopic surgical PD catheter insertions were introduced to the PD service. Laparoscopic procedures were gradually introduced across multiple National Health Service (NHS) and independent sector sites from late 2020. Fluoroscopically guided percutaneous PD catheters insertions supported by a dedicated interventional radiologist were introduced to the PD service in 2021. After the appointment of a Home Therapy Lead, a nephrology-led service for percutaneous PD catheter insertion using an ultrasound-guided technique was developed in the Northern Trust. For the treatment, patients are considered on case-by-case basis in order to select the appropriate technique and setting for PD catheter insertion and ensure that it is in line with recommendations from the International Society for Peritoneal Dialysis [17].

### 2.3. Technical Details

#### 2.3.1. Laparoscopic Surgical PD Catheter Insertion

Procedures were performed in theatre under general anaesthesia. After initial access of a camera port, introduction of the PD catheter was performed under vision via either a 16Fr Peel-Away^®^ Sheath Introducer (Cook Medical, Hitchin, UK) or eight-millimetre laparoscopic port (Endopath ^®^ Xcel, Ethicon, London, UK, Ethicon Endo-Surgery). A rectus sheath tunnel was created to angle the PD catheter toward the pelvis [17]. Further laparoscopic ports were only used if adhesiolysis, PD catheter repositioning, or suture fixation were deemed to be required.

#### 2.3.2. Percutaneous PD Catheter Insertions

Fluoroscopically guided procedures were performed in an interventional radiology suite in the Regional Centre—using ultrasound to identify and avoid injury to vessels and viscera and fluoroscopy to observe safe needle entry by the flow of injected contrast around loops of bowel. Ultrasound-guided procedures were performed in a procedure room in the Northern Trust Renal Unit. In both approaches, a guidewire was directed toward the pelvis, and after dilating up a track, the PD catheter was inserted via a 16Fr Peel-Away^®^ Sheath Introducer (Cook Medical).

#### 2.3.3. Peri-Procedural Care

Pre-procedure, patients received Methicillin-resistant Staphylococcus Aureus (MRSA) and Methicillin-sensitive Staphylococcus Aureus (MSSA) decolonisation and bowel preparation. Intravenous Teicoplanin was used for antibiotic prophyalxis. After insertion, PD catheters were subcutaneously tunnelled to the desired exit site using a 16Fr drain spike from a high vacuum wound drainage system (Medinorm ^®^, Summit Medical, Cotswolds, UK). All catheters were tested with Heparanised Saline (5000 units/500 mL) to confirm good inflow and outflow. Heparinised Saline was left in the peritoneal cavity to try to reduce the risk of omental wraps and fibrin plugs. Exit sites were dressed with a Biopatch (Ethicon), sterile gauze, and 3M™ Tegoderm™ Transparent Film Dressings. Dressings were left intact for 5 days.

### 2.4. Data Collection and Analysis

All patients who had a PD catheter inserted during the calendar year 2021 were included and followed up for at least one-year. Data were collected from a prospectively collected Regional audit database (Belfast Trust Audit and Quality Improvement Reference 6354). Demographic details, primary renal disease, previous RRT modality, insertion technique, patency, and outcome data were collected. Primary patency was defined as a functioning PD catheter that did not require removal, replacement, or requirement for intervention because of flow dysfunction or drain pain [17]. Primary assisted patency was defined as a functioning PD catheter that did require manipulation because of flow dysfunction or drain pain. Secondary patency was defined as a functioning PD catheter after the previous catheter needed to be replaced because of flow dysfunction or drain pain. Loss of patency is censored for death, transplant, infection, or transfers to HD because of inadequate dialysis, psychosocial reasons, or medical problems [17]. Time-to-event survival analysis was used to evaluate primary, primary assisted, and secondary patency of peritoneal dialysis catheters. A Kaplan–Meier curve was then generated to graphically display patency survival. Statistical analysis was performed using R version 4.2.2.

## 3. Results

In 2020, there were 30 patients who had a PD catheter inserted in NI. This is similar to the annual number of PD catheter insertions performed in 2018 and 2019 prior to the COVID-19 pandemic [12].

After service reconfiguration in 2021, there were 66 patients per annum (summarised in Table 1) who had a PD catheter inserted in Northern Ireland—a two-fold increase.

### 3.1. Introduction of a Wider Portfolio of PD Catheter Insertion Techniques

A laparoscopic technique was used for PD catheter insertion in 41 patients, and in 24 patients, a percutaneous approach was used (19 fluoroscopically guided and 5 ultrasound-guided). Only one patient had a PD catheter inserted using an open technique under general anaesthesia due to their preference of the available surgeon.

### 3.2. Offering PD to a Wider Group of Patients, Including Those Considered High-Risk for General Anaesthesia and with Extensive Previous Surgery

Consistent with an initial policy of selecting more anaesthetically high-risk patients for local anaesthetic procedures, people having a percutaneous PD catheter insertion were significantly older than those having laparoscopic PD catheter insertion [median age 76 (range 37–88) vs. 56 (range 18–84), *p* < 0.0001]. Patients having a percutaneous PD insertion also had less previous abdominal surgery than those having a laparoscopic PD catheter insertion (25%, 6/24, vs. 22/41, 22/41 *p* = 0.05). In the laparoscopic PD catheter insertion group, five patients had a history of previous midline laparotomy. Six patients in the laparoscopic PD catheter insertion group required laparoscopic adhesiolysis. Two patients underwent a simultaneous ventral hernia repair at the time of laparoscopic PD catheter insertion.

### 3.3. Offering Urgent and Early Start PD

There were six patients that underwent laparoscopic PD catheter insertions in an emergency theatre in the Regional Centre. In two emergency cases, acute inpatient PD was started—beginning on day one and day two post-operation [18]. In another two emergency cases, early inpatient PD was started—beginning on day four and day five post-operation. In the other two cases, early inpatient PD was anticipated but not required.

### 3.4. Performing Elective PD Catheter Insertions in Multiple NHS and Independent Sector Sites

In the other 35 patients who underwent elective laparoscopic PD catheter insertions, thirteen procedures were performed in other NHS sites (eleven in separate trusts), thirteen were performed in the independent sector under a NHS contract, and only nine were performed in the Regional Centre. Combined with the five patients receiving ultrasound-guided percutaneous PD catheter insertions in their local unit, nearly half (48%, 29/60) of PD catheters inserted electively in 2021 were in smaller elective hubs rather than the Regional Centre.

### 3.5. Outcomes

**Figure 1:**
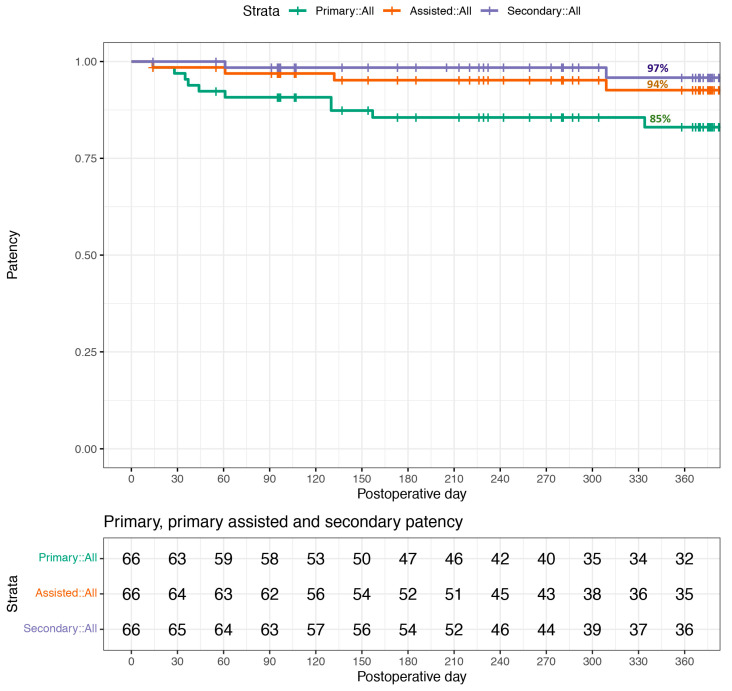
Primary, primary assisted, and secondary patency for peritoneal dialysis catheter insertions in 2021 (*n* = 66). Loss of patency is censored for death, transplant, infection, or transfers to HD because of inadequate dialysis, psychosocial reasons, or medical problems [17].

Ten patients needed an additional manipulation procedure (*n* = 6), required PD catheter replacement (*n* = 3), or declined intervention for catheter dysfunction (*n* = 1)—giving a primary patency rate of 85% (56/66). After accounting for six patients that required a manipulation procedure, the primary assisted patency rate was 94% (62/66). After further accounting for two patients that required replacement catheters, the secondary patency rate was 97% (64/66) (Figure 1). In addition to the patient who declined intervention, a patient with a BMI of 43 and extensive pelvic surgery had persistent PD catheter flow dysfunction even after PD catheter replacement. Overall outcomes at one year after follow-up are summarised in Table 2.

## 4. Discussion

This study demonstrates how, through a service reconfiguration bundle, we were able to double the number of PD catheter insertions and expand access to PD for patients within our region. This increase was rapidly achieved within a single year in 2021. This improvement was sustained with 57 PD catheter insertions performed in 2022. The increased initiation of PD along with kidney transplant numbers returning to pre-pandemic levels has allowed the region to stabilise and even reduce the overall number of patients receiving in-centre HD [12,19]. Whilst, 1-year post insertion, the number of those remaining on PD was lower than other reported studies (55%). This is due to the high rate of transplantation in our cohort (21%) [20]. The higher death rate of 11% also reflects the frailty of the population accessing the service via percutaneous insertion in particular, and deaths appeared unrelated to catheter insertion. Though limited to a single region, our results and experience suggest potential strategies that can lead to a service that increases access to PD for patients with ESKD.

### 4.1. Staffing Expertise and Training

Developing specialty interests in PD catheter insertion among specific members of the surgical and interventional radiology team has assisted in establishing a change in practice. Having champions for PD and improving access to catheter insertion increased referral by nephrologists. Often if there are perceived barriers (such as delays in catheter insertion due to waiting times), it can affect both clinicians’ and patients’ preference of RRT modality [21]. Furthermore, protected time for clinicians to consolidate skills and work collaboratively can increase service quality and promote innovation [22].

Unlike other jurisdictions, in the UK, it is not a requirement for nephrology specialty trainees to become proficient in ultrasound-guided PD catheter insertion [23]. In some countries, most PD catheter insertions are performed by nephrologists, leaving complex cases for surgical insertion—creating a true hub and spoke model [24]. Due to the small annual number of PD catheter insertions (some of which are not suitable for an ultrasound-guided approach) in NI and other small regions, it can be difficult to train and maintain the expertise of nephrologists [25]. This requires healthcare providers to enable staff (with both time and investment) to visit and train in larger centres to acquire these skills but also strategic region-wide planning for service delivery to train personnel in areas of most need [26]. This approach allowed the Home Therapy Lead to set up a nephrology-led insertion service in an area of particular need in NI.

### 4.2. Introducing a Wider Portfolio of PD Catheter Insertion Techniques

Through diversifying catheter insertion techniques and developing staff skills in new procedural competencies, we have been able to offer a variety of clinical pathways for patients to obtain access to PD. The COVID-19 pandemic, coupled with the significant waiting list pressures in the National Health Service, has prompted many to reconsider clinical pathways [27]. Clinical services need to have in-built resilience so that they can adapt to changing pressures and demands. Having multiple different routes for patients to acquire PD catheter insertion has helped us to reduce waiting times [28].

Our results demonstrate not only an increased number of PD catheter insertions but that a wider range of patients were able to benefit from PD. Higher risk patients had successful PD catheter insertions using percutaneous techniques that avoid general anaesthetic. In previous years, these patients may have been restricted to HD or conservative care. Many of these higher risk patients have cardiac disease associated with poor tolerability of intradialytic haemodynamic instability experienced during HD [29]. Hence, the introduction of the percutaneous PD catheter insertion technique not only extends patient choice but offers some patients a more optimal RRT modality, given their comorbidities [30]. This has also been paired with an increase in the capacity for assisted PD for frailer patients and the tailoring of dialysis prescriptions to their specific requirements [31].

The adoption of a laparoscopic surgical approach allowed patients with extensive previous abdominal surgery to successfully commence PD [32]. Surgical and percutaneous approaches have similar outcomes [33,34], but a laparoscopic approach facilitates patients with relative contraindications to PD (abdominal hernia and adhesions) to be considered, with simultaneous hernia repair or adhesiolysis again expanding access to more patients.

### 4.3. Being Able to Facilitate Urgent and Early Start PD

Laparoscopic catheter insertion requires reduced recovery time prior to initial PD treatment—expanding the potential of PD use in patients requiring urgent dialysis [35]. In total, six patients had emergency catheter insertion, though only four subsequently went on to receive urgent or early PD. This could allow urgent/early PD and facilitates patients who have experienced an unexpected rapid deterioration in their kidney function to receive their first choice of dialysis modality rather than having to default to HD [35]. This is particularly important given the observably low rates of successful conversion from HD to PD and increased mortality in these patients compared to those who initially begin with PD [36]. It also facilitated the sparing of vascular access options for younger patients who presented with ESKD and face a lifetime of RRT [37].

### 4.4. Use of Elective Hubs

The ability of surgical staff to travel to other hospital sites away from the regional unit allowed the use of theatre schedules and staff previously unavailable to nephrology services. In 2021, nearly half of the procedures involving the insertion of elective PD catheters were performed in smaller elective hubs rather than the regional unit—where access to elective theatres can be limited due to high demand and complex cases. These measures not only enable the increased delivery of PD catheter insertions but offer a more diverse and resilient clinical pathway and are less susceptible to disruption from factors such as winter bed pressures. This model, where specialised teams travel to provide elective surgical procedures, has been highlighted by many reports as a method to reduce elective waiting lists [38].

### 4.5. Future Considerations

Patient groups and international bodies are advocating for the expansion of home therapies to deliver more tailored therapy to patients with ESKD and combat the globally rising numbers of patients receiving HD [39]. Increasing the amount of patients using PD as their RRT has effects on wider nephrology services. With an increase in access to PD catheter insertion, there also needs to be an increase in staff with expertise regarding PD. Community support and enhanced systems for the ongoing delivery of care for patients with PD need to be developed. This will require reprioritisation of investment in nephrology services and careful workforce planning to ensure sustainable delivery of PD [39].

## 5. Conclusions

This study demonstrates how, through a service reconfiguration bundle, we were able to transform our PD service in Northern Ireland—effectively doubling our annual incident PD population and widening access to older and frailer patients who represent a large proportion of the at-risk CKD population. Additionally, we have highlighted how staff allocation and training, along with flexible models of delivery, can expand access to PD as a RRT option for patients.

## Figures and Tables

**Table 1:** Demographics of patients undergoing peritoneal dialysis catheter insertions in 2021 (*n* = 66).

Variable	Median (Range) or *n* (%)
Age	67 (18–88)
Sex	
Female	23 (35%)
Male	43 (65%)
BMI	26 (19–43)
Primary renal disease	
Diabetic nephropathy	20 (30%)
IgA nephropathy	5 (8%)
Polycystic kidney disease	4 (6%)
Focal segmental glomerulosclerosis	3 (5%)
Other (including unknown)	34 (52%)
Pre-procedure glomerular filtration rate	10 (5–15)
Switching from haemodialysis	11 (17%)

**Table 2:** Outcomes of patients undergoing peritoneal dialysis catheter insertions in 2021 (*n* = 66).

Outcome	*n* (%)
Completed 1-year follow-up on Peritoneal Dialysis	36 (55%)
Transplanted	14 (21%)
Died *	7 (11%)
Switch to Haemodialysis **	9 (14%)

* Causes; upper gastrointestinal bleed (day 7), withdrawal from dialysis (day 55), stroke (day 213), myocardial infarction (day 220), gastrointestinal perforation (day 242), pulmonary oedema (day 281), and ischemic colitis (day 291). ** Reasons; Psychosocial reasons (*n* = 2), pleural leak (*n* = 2), flow dysfunction (*n* = 2), inadequacy (*n* = 1), diverticular perforation (*n* = 1), and PD peritonitis (*n* = 1).

## Data Availability

The data presented in this study are available on request from the corresponding author. The data are not publicly available due to privacy.
